# Shedding Light on Acne Scars: A Comprehensive Review of CO2 vs. Erbium-Doped Yttrium Aluminium Garnet (Er:YAG) Laser Therapy

**DOI:** 10.7759/cureus.57572

**Published:** 2024-04-03

**Authors:** Soham R Meghe, Arshiya Khan, Shivani D Jangid, Bhakti Sarda, Nitya Vangala, Vikrant Saoji

**Affiliations:** 1 Dermatology, Jawaharlal Nehru Medical College, Datta Meghe Institute of Higher Education and Research, Wardha, IND

**Keywords:** scar improvement, treatment comparison, er:yag laser, co2 laser, laser therapy, acne scars

## Abstract

Acne scars pose a significant cosmetic concern and can have a profound impact on individuals' self-esteem and quality of life. Laser therapy has emerged as a promising treatment modality for improving the appearance of acne scars by promoting collagen remodeling and tissue regeneration. This comprehensive review compares two commonly used laser modalities, CO2 and erbium-doped yttrium aluminum garnet (Er:YAG), focusing on their mechanisms of action, efficacy, safety profiles, and patient outcomes. While CO2 lasers offer deeper tissue penetration and the potential for more significant improvement in severe acne scars, Er:YAG lasers provide a gentler approach with a lower risk of post-inflammatory hyperpigmentation. Recommendations for clinical practice include tailoring treatment approaches to individual patient characteristics, educating patients about treatment expectations and post-treatment care, considering combination therapies for enhanced outcomes, and implementing regular follow-up care. Areas for further research include long-term outcome studies, investigation of laser therapy in ethnically diverse populations, exploration of combination therapies, and evaluation of emerging laser technologies. This review aims to provide clinicians and patients with valuable insights to inform treatment decisions and optimize outcomes in managing acne scars.

## Introduction and background

Acne scars are lasting reminders of past acne breakouts, characterized by alterations in the skin's texture and appearance [1]. These scars can manifest in various forms, including depressions, raised areas, and discolorations, and they often result from the inflammatory response to acne lesions. Acne scars not only affect one's physical appearance but can also have a significant impact on self-esteem and quality of life [2].

Laser therapy has emerged as a promising treatment modality for acne scars due to its ability to target and remodel scar tissue precisely. Unlike topical creams or traditional surgical methods, laser therapy offers a non-invasive approach with minimal downtime and predictable outcomes [3]. The controlled delivery of laser energy allows for precise tissue ablation and collagen remodeling, leading to smoother and more even skin texture [4]. This review aims to comprehensively compare two commonly used laser modalities for treating acne scars: carbon dioxide (CO2) and erbium-doped yttrium aluminum garnet (Er:YAG) lasers. By examining the mechanisms of action, efficacy, safety profiles, and patient outcomes associated with each laser type, this review aims to assist clinicians and patients in making informed decisions regarding treatment selection. Additionally, this review will explore the factors influencing treatment choice and discuss emerging technologies and future directions in laser therapy for acne scars.

## Review

Understanding acne scars

Types of Acne Scars

Atrophic scars: Atrophic scars are indented scars that form when the skin heals below the normal layer of skin tissue. These scars are characterized by a missing portion of tissue that the body does not fill in, leading to their crater-like appearance. Atrophic scars are subdivided into three main categories: ice pick scars, boxcar scars, and rolling scars. Ice pick scars are narrow, deep, and sometimes V-shaped, accounting for most atrophic scarring cases. Boxcar scars have flat bottoms and defined borders, resembling small potholes, while rolling scars lack distinct edges and are more expansive, typically found on the cheeks [5,6]. The causes of atrophic scarring include severe acne, chickenpox, injury, or surgical cosmetic procedures like mole removal. While completely removing ancient atrophic scars can be challenging, various treatments can help reduce their appearance. Treatment options include chemical peels, fillers, subcision, dermabrasion, microneedling, and more, which can help decrease the indenting appearance of these scars and re-level the skin. Consulting a qualified dermatologist for tailored treatment recommendations is essential to address atrophic scars effectively [5,6].

Hypertrophic scars: Hypertrophic scars are characterized by excessive collagen deposition, resulting in raised scars that do not extend beyond the original wound boundaries. These scars typically develop after thermal or traumatic injuries involving the deep layers of the dermis, often forming at sites of pimples, body piercings, cuts, or burns. They are red to brown, thick, and may be itchy or painful, improving over one to two years but causing distress due to appearance or itching. Mechanical tension on a wound is a leading cause of hypertrophic scar formation, and individuals with Ehlers-Danlos syndrome may have an inherited tendency towards these scars [7,8]. Treatment options for hypertrophic scars include pressure garment therapy, corticosteroid injections, silicone gel sheeting, and laser therapy. Early scars can be managed with pressure and massage, while ongoing hypertrophy may require corticosteroid injections. Silicone gel sheeting and laser therapy have been studied for their effectiveness in treating hypertrophic scars, with varying degrees of success reported. Surgical revision may be considered after one year of scar development [7,8]. It is important to note that hypertrophic scars differ from keloid scars, as they are confined within the original wound area and are more likely to regress and resolve compared to keloids. While hypertrophic scars are more common and can occur in all races and ages, keloids are less common and have a higher recurrence rate. Understanding the differences between these two types of raised scars is crucial for appropriate diagnosis and treatment planning [7,8].

Causes of Acne Scars

Inflammation: Inflammation plays a pivotal role in the genesis of acne scars. The onset of acne vulgaris prompts an inflammatory cascade within the skin, culminating in diverse scar types. Inflammatory acne, typified by red, swollen, and painful lesions, may progress to deep-seated nodules, pustules, or cysts harboring bacteria, pus, dead skin cells, and sebum. Rupture or manipulation of these inflamed lesions exacerbates skin damage, fostering scar formation. The inflammatory milieu in acne entails a sophisticated interplay of factors, encompassing altered wound healing responses, infiltration of immune cells, and dysregulated collagen turnover. This intricate interplay eventuates in the development of atrophic scars, hypertrophic scars, or keloids. Effective management of inflammatory acne is imperative to mitigate scarring risk, necessitating a spectrum of interventions from over-the-counter skincare formulations to professional therapeutic modalities like laser therapy, dermabrasion, and topical or systemic medications [9,10].

Delayed healing: Many factors elucidated in the provided sources can influence the protracted healing process of acne scars. Stress decelerates the body's innate healing mechanisms, necessitating a heightened intake of specific nutrients such as vitamin C. Furthermore, skin picking, excessive exfoliation, and failure to seek dermatological guidance can protract scar resolution. Inadequate sleep quality compromises the body's reparative processes, underscoring the importance of employing nocturnal skincare regimens that facilitate skin rejuvenation. Delaying intervention for active acne breakouts and scars may yield short-term cost savings but can engender prolonged distress. Timely initiation of treatment with efficacious skincare formulations containing retinoids can ameliorate acne outbreaks and ameliorate scar appearance. For individuals necessitating more aggressive scar remediation strategies, consulting with a qualified healthcare provider is advisable to explore tailored options such as laser skin resurfacing or surgical techniques like excision and subcision for enduring scar eradication or amelioration [11-13].

Collagen Disruption

Collagen disruption encompasses various facets, each holding significance across different contexts. A study highlights a newfound mechanism of collagen fibril disruption, revealing molecular denaturation at specific sites. This sheds light on the resilience of collagen fibrils and its implications for tissue mechanics, offering insights into the underlying toughness of collagen structures [14]. Further exploration into human collagen degradation pathways underscores the importance of comprehending these processes. Such understanding holds promise for potential therapeutic interventions in conditions like atherosclerosis, where collagen degradation is pivotal [15,16]. Moreover, disruptions in collagen homeostasis are intricately linked to age-related changes. Modulating these disruptions can profoundly affect collagen synthesis and tissue remodeling [17]. Collectively, these findings emphasize the complexity of collagen disruption and its far-reaching implications. From maintaining tissue integrity to influencing disease progression, understanding collagen disruption opens avenues for innovative therapeutic strategies to preserve and enhance tissue function.

Overview of CO2 laser therapy

Mechanism of Action

The mechanism of action underlying CO2 laser therapy is predicated upon light emission at a specific wavelength of 10,600 nm, which exhibits strong absorption by water molecules, the primary chromophore in skin tissue [18,19]. This laser energy is absorbed within the superficial 20-30 µm layer of the skin, facilitating meticulous tissue ablation with minimal thermal injury compared to conventional continuous wave lasers [19]. Central to the process is the principle of selective photothermolysis, wherein laser pulses lasting less than one millisecond selectively vaporize tissue with an energy fluence of approximately 5 J/cm2, resulting in the creation of a zone of thermal necrosis that concurrently cauterizes small blood vessels and lymphatics, thereby diminishing the risk of scarring [18,19]. CO2 lasers operate by completely removing the epidermis and partially ablating the dermis, thereby instigating wound remodeling and provoking the synthesis of fresh collagen and elastin fibers, promoting enhanced skin firmness and tautness [18]. The technological framework underlying CO2 lasers encompasses high-powered pulsed lasers in tandem with optomechanical flash scanners. These facilitate precise energy delivery to targeted tissues, fostering effective skin resurfacing and scar revision [18,19]. In essence, the mechanism of action inherent in CO2 laser therapy revolves around meticulous tissue ablation, promotion of collagen synthesis, and orchestration of wound healing processes to achieve desired dermatological outcomes.

Efficacy

Fractional CO2 laser treatment has demonstrated notable efficacy across various studies for diverse conditions. Research indicates that this modality effectively ameliorates symptoms associated with genitourinary syndrome of menopause, including vaginal pH imbalance, dryness, dyspareunia, burning sensations, and itching, thereby providing significant short-term relief [20,21]. Furthermore, CO2 laser therapy exhibits beneficial effects in alleviating symptoms linked to vulvovaginal atrophy in postmenopausal women, underscoring its efficacy in managing these conditions [22]. Moreover, the combination of fractional CO2 laser with stromal vascular fraction (SVF) has been shown to enhance therapeutic outcomes compared to using the laser alone, particularly in treating skin scars such as burns and acne scars. This combination therapy substantially improves skin parameters and augments patient satisfaction [23]. The versatility of CO2 laser therapy extends to addressing cosmetic skin concerns, including fine lines, wrinkles, acne scars, sunspots, and age spots, rendering it a valuable treatment modality for skin rejuvenation and managing diverse dermatological issues [24].

Side Effects and Risks

After CO2 laser treatment, there is a risk of infection, which can stem from various sources, including bacterial, viral, yeast, or fungal organisms. Prompt intervention with topical and systemic antibiotics is imperative in the event of an infection [25-27]. Skin peeling often ensues as a common side effect post-CO2 laser treatment, characterized by the shedding of the outer layer of the skin as part of the natural healing process [25]. Skin redness is another typical side effect following CO2 laser resurfacing, which may persist during healing [25,28]. Moreover, alterations in skin tone, encompassing hyperpigmentation or hypopigmentation, can manifest post-treatment, requiring time to resolve or potentially becoming permanent in some instances [26]. Although scarring is generally infrequent with CO2 laser resurfacing, there remains a risk of hypertrophic or keloid scarring, particularly among individuals predisposed to such conditions [26]. Additionally, post-treatment, small white bumps or cysts known as milia may surface on the skin, although they typically resolve spontaneously or can be removed if they pose a nuisance [29]. Ectropion, characterized by the outward turning of the lower eyelid, is a rare but potential complication associated with CO2 laser therapy [27]. Though rare, dental enamel injuries have been documented as a possible adverse effect of CO2 laser treatment [27]. Furthermore, unusual hypersensitivity reactions may occur following CO2 laser resurfacing, necessitating vigilant monitoring and appropriate management [27]. Lastly, some individuals may experience delayed wound healing after CO2 laser treatment, underscoring the importance of meticulous postoperative care to foster optimal healing outcomes [29].

Patient Selection

Before proceeding with CO2 laser treatment, it is crucial to comprehensively evaluate the patient's skin type and condition, considering factors such as the degree of photodamage, pigmentation issues, the presence of wrinkles, scarring, and overall skin health [30]. Additionally, patients should be encouraged to maintain realistic expectations regarding the procedure's outcomes. Unrealistic expectations, such as anticipating the complete eradication of wrinkles or scars, can significantly impact patient satisfaction and post-treatment experience [31]. A thorough assessment of the patient's medical history is imperative, encompassing past laser resurfacing treatments, recent use of isotretinoin, and any history of skin conditions such as acne vulgaris or cutaneous carcinomas [32]. Moreover, identifying contraindications is essential to prevent complications. This includes screening for active cutaneous bacterial infections, herpes simplex, connective tissue disorders, prior deep chemical peels or dermabrasion, Fitzpatrick skin phototypes 5 or 6, and HIV or hepatitis C infections [31]. Preoperative preparation plays a vital role in optimizing patient outcomes. Initiating patients on a daily skin conditioning regimen several weeks before the procedure can enhance skin health and minimize risks associated with CO2 laser resurfacing [30]. Subsequently, providing appropriate postoperative care is crucial for reducing the risk of infection and managing potential side effects such as redness, hyperpigmentation, or hypopigmentation, thereby facilitating optimal healing [31]. Furthermore, understanding potential complications, including scarring, prolonged erythema, hypopigmentation, or infections, is paramount for proper patient selection and management during and after CO2 laser resurfacing [33]. By adhering to these considerations and protocols, clinicians can ensure safe and effective treatment outcomes for patients undergoing CO2 laser therapy.

Overview of Er:YAG laser therapy

Mechanism of Action

The mechanism of action underlying Er:YAG laser therapy revolves around tissue ablation, facilitated by its heightened affinity for water. Emitting a wavelength of 2940 nm, the Er:YAG laser exhibits a superior attraction to water molecules compared to the CO2 laser, thereby enabling precise tissue ablation while minimizing thermal damage. The laser's absorption properties dictate the depth of penetration and dispersion of thermal energy, with the energy dissipated as heat to facilitate tissue remodeling. Unlike the CO2 laser, the Er:YAG laser exerts minimal thermal effects on collagen shrinkage, thus mitigating the risk of pigmentary alterations and scarring. With its shallow penetration depth and abbreviated pulse duration, the Er:YAG laser achieves tissue ablation with minimal collateral thermal damage, rendering it efficacious for skin rejuvenation and treating various dermatological conditions [34,35].

Efficacy

The effectiveness of Er:YAG laser therapy has been substantiated through numerous studies spanning diverse medical disciplines. In the realm of urinary conditions, research revealed that 75% of patients witnessed some degree of improvement following Er:YAG laser treatment, with results enduring for up to 48 months [36]. Furthermore, Er:YAG laser resurfacing in dermatology and dentistry has emerged as a potent therapeutic tool for addressing various cutaneous concerns and dental procedures. Its applications include skin rejuvenation, scar mitigation, and the treatment of hypersensitive dentin, offering multifaceted benefits [37-40]. Moreover, the utilization of Er:YAG lasers in treating peri-implantitis has demonstrated superiority over traditional mechanical debridement techniques in reducing pocket depth and gingival recession, underscoring its clinical efficacy in this specific application [4]. Er:YAG laser therapy has consistently exhibited efficacy across various medical contexts, positioning it as a valuable treatment modality for various conditions.

Side Effects and Risks

**Figure 1:**
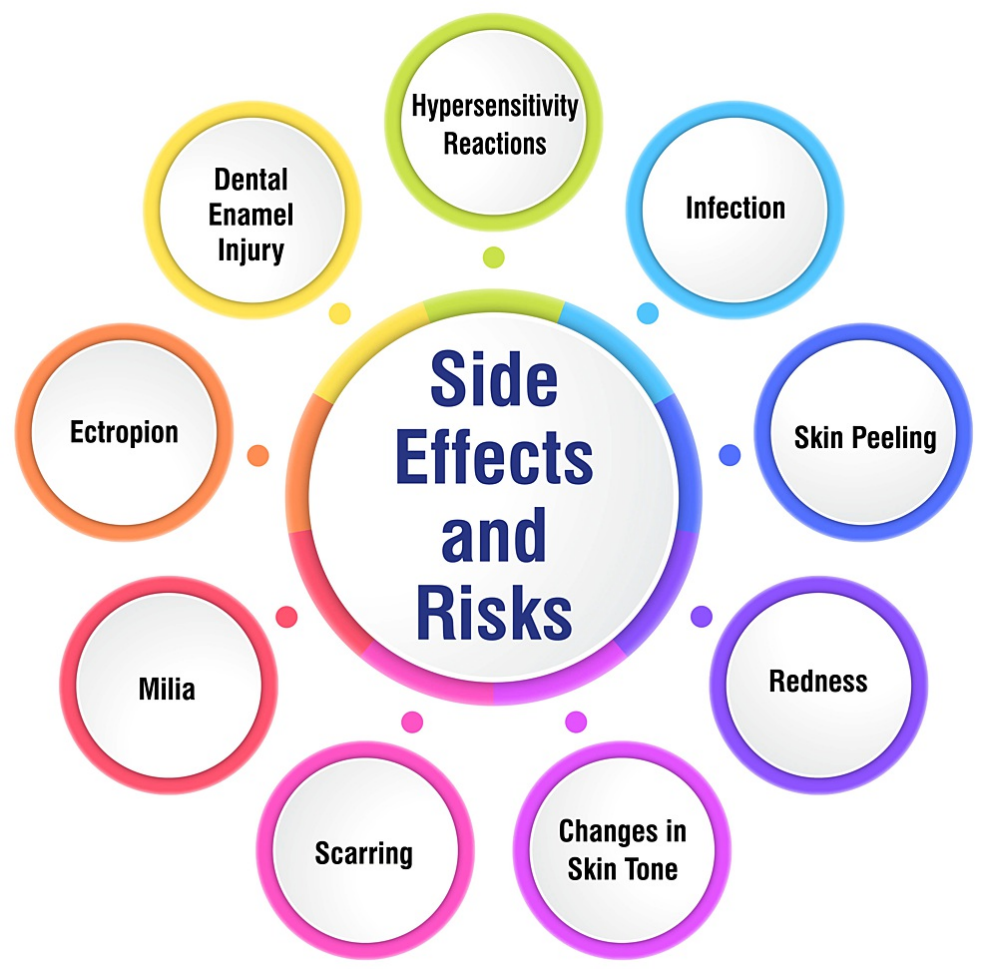
Side effects and risks Corresponding author Soham Meghe created this figure.

Patients considering Er:YAG laser therapy should be cognizant of the potential side effects and risks associated with the procedure to make informed decisions regarding their treatment. Common mild side effects of Er:YAG laser treatment may include milia development, acne exacerbation, and contact dermatitis. Moderate effects can involve localized infections such as herpes simplex or impetigo, prolonged redness, and post-inflammatory hyperpigmentation. While severe side effects are rare, they may encompass fibrosis, hypertrophic scarring, and the occurrence of ectropion, characterized by the outward rolling of the eyelid margin [41]. Moreover, precautions should be exercised for patients with specific conditions such as a propensity toward keloid or hypertrophic scar formation, diminished numbers of adnexal skin structures, or a history of prior ionizing radiation to the skin [41]. To ensure optimal treatment outcomes, patients must comprehend these potential side effects and risks associated with Er:YAG laser therapy. By being well-informed, patients can actively participate in their treatment decisions and work collaboratively with healthcare professionals to minimize risks and achieve the desired results. Side effects and risks are shown in Figure 1.

Patient Selection

**Figure 2:**
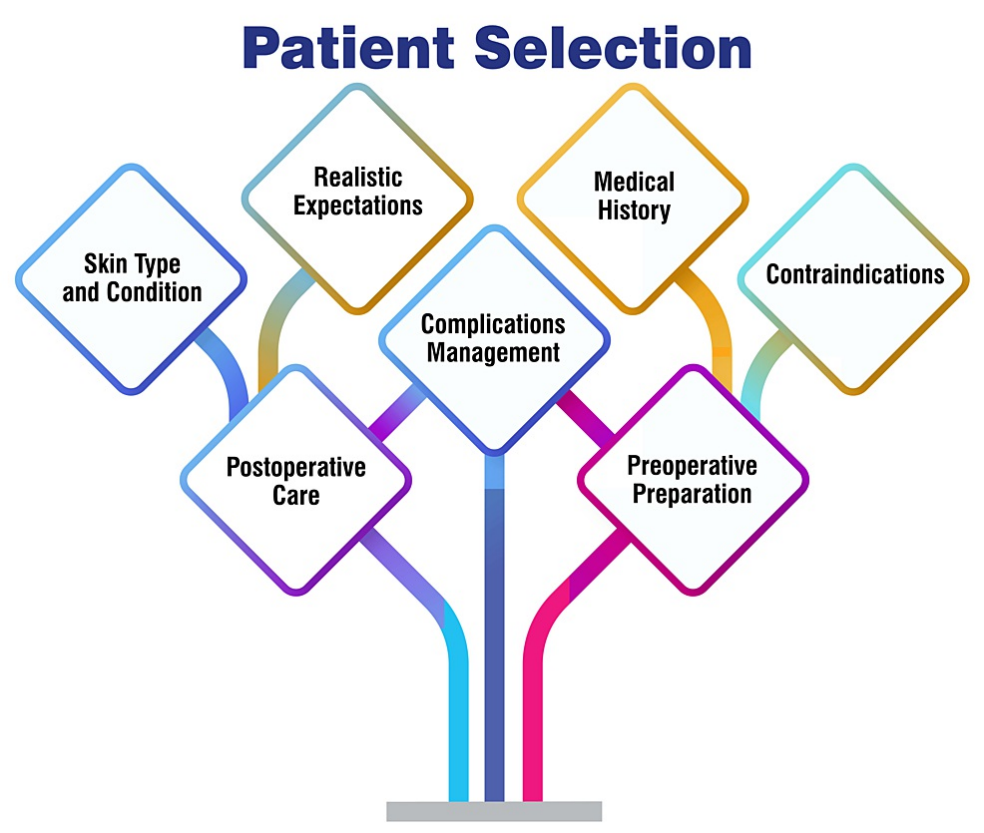
Patient selection Corresponding author Soham Meghe created this figure.

Patient selection is pivotal in optimizing Er:YAG laser treatment outcomes. Studies have underscored the efficacy of this modality for addressing stress urinary incontinence (SUI) in women, particularly those with mild to moderate SUI who have not responded favorably to alternative treatments. In the context of SUI, intraurethral Er:YAG laser therapy has exhibited a 77% success rate in symptom improvement, accompanied by minimal adverse effects such as mild discomfort and urinary infections [42,43]. Furthermore, Er:YAG laser therapy has demonstrated promise in the treatment of snoring, with the non-invasive NightLase treatment (Fotona, Ljubljana, Slovenia) utilizing a 2940 nm Er:YAG laser proving effective in reducing snoring among adult patients [44]. When selecting patients for Er:YAG laser therapy, factors to consider include the type and severity of the condition being treated, previous responses to treatments, and potential contraindications such as pelvic organ prolapse. Patient selection is shown in Figure 2.

Comparative analysis of CO2 and Er:YAG laser therapy

Treatment Outcomes

A systematic review and meta-analysis have revealed highly effective treatment outcomes of fractional CO2 laser therapy for depressed acne scars. Studies indicate that ultra-pulse CO2 fractional laser treatment yields favorable results in patients with depressed scars by stimulating collagen production, refining skin texture, and expediting the healing process. This laser therapy is distinguished by its reduced trauma, rapid healing, minimized complications, and commendable efficacy in addressing pitted acne scars [45]. Furthermore, a comparative study between fractional CO2 and Er:YAG lasers unveiled that fractional CO2 laser treatment significantly outperformed Er:YAG laser therapy for acne scars, evidenced by higher subjective satisfaction and physicians' assessment scores [46]. These findings underscore the advantageous outcomes of fractional CO2 laser treatment for depressed acne scars, underscoring its efficacy and potential as a preferred modality for acne scar management.

Safety Profiles

The safety profiles of laser therapy, particularly low-level laser therapy (LLLT), are generally favorable, with only minor adverse effects reported. Laser therapy is considered safe for clinical applications when conducted by proper safety protocols and guidelines. However, healthcare professionals must exercise caution and attentiveness to safety measures to mitigate risks associated with laser therapy. Adherence to laser safety guidelines, utilization of appropriate protective gear, and ensuring that laser devices comply with FDA safety standards are fundamental steps in maintaining a safe environment for both practitioners and patients during laser treatments [47]. Additionally, a Laser Safety Officer (LSO) in clinics utilizing lasers is crucial to overseeing and enforcing safety protocols, conducting routine safety assessments, and ensuring appropriate management of laser-related injuries or incidents [48]. While laser therapy can provide significant therapeutic benefits, prioritizing laser safety is essential to mitigate potential adverse effects and safeguard the well-being of individuals undergoing laser treatments.

Cost Considerations

The cost considerations for CO2 laser resurfacing can vary significantly, influenced by several factors, including the type of treatment (ablative or fractional), geographical location, the credentials and expertise of the dermatologist or surgeon, the number of sessions required, and the size of the treatment area. On average, CO2 laser resurfacing procedures may range around $2750, with some individuals reporting expenses of up to $6100 for the treatment [24]. Typically, cosmetic procedures like CO2 laser resurfacing are not covered by insurance, although there may be coverage for medically necessary treatments such as skin cancer removal. It is advisable to seek consultation with a reputable, board-certified dermatologist or plastic surgeon to gain clarity on cost implications, potential insurance coverage, and expected outcomes of CO2 laser resurfacing tailored to individual needs and circumstances.

Patient Satisfaction

Patient satisfaction with laser therapies, mainly CO2 and Er:YAG lasers, appears notably high based on the provided sources. For example, a study investigating combined CO2/Er:YAG laser treatment for neck resurfacing documented a significant enhancement in skin texture and color, leading to high overall patient satisfaction. Moreover, no adverse effects were reported, such as hypopigmentation or scarring [49]. Similarly, fractional Er:YAG resurfacing for photo-aged skin yielded positive patient satisfaction ratings seven days and two months post-treatment [50]. Furthermore, Er:YAG laser therapy for burn scar treatment resulted in more than 90% of patients reporting improvement and expressing high satisfaction levels [51]. These findings collectively underscore the efficacy of laser therapies in heightening patient satisfaction by addressing a spectrum of skin concerns, including aging, scarring, and skin texture irregularities.

Factors influencing treatment selection

Scar Severity

The severity of scars can vary widely, encompassing various types such as fine-line, atrophic, hypertrophic, and contracture scars. Fine-line scars typically start slightly raised but tend to flatten and fade over time without treatment. Conversely, atrophic scars may manifest as small indentations in the skin or larger sunken marks, often stemming from conditions like acne or chickenpox. Hypertrophic scars present as raised, firm, and smooth protrusions, expanding beyond the boundaries of the original wound and potentially causing discomfort or limiting movement. Unlike fine-line scars, hypertrophic scars do not typically diminish in size or visibility without intervention. Contracture scars are tight and may impede movement or induce pain, commonly emerging following burn injuries [52]. Furthermore, accurately predicting scar severity postoperatively is essential for determining suitable treatment approaches. Factors such as body mass index (BMI), the type of surgical procedure, and associated symptoms like itching, pain, adhesion, tightening, or swelling play pivotal roles in evaluating scar severity [53]. Additionally, quantitative scales for clinical scar assessment have been developed in studies to objectively evaluate scars and assess the effectiveness of preventive or therapeutic regimens [54]. Moreover, severe cutaneous adverse reactions (SCAR) denote critical skin responses to medications that can pose life-threatening risks. These reactions encompass severe conditions such as Stevens-Johnson syndrome and toxic epidermal necrolysis [55].

Skin Type and Sensitivity

Sensitive skin can manifest in various ways, presenting symptoms such as stinging, itching, burning, redness, dryness, scaling, peeling, bumps, and hives [56]. Typically, four common types of skin sensitivity are identified: allergic sensitivity, breakout-prone sensitivity, heat-activated sensitivity, and dry/dehydrated sensitivity [57]. Allergic sensitivity involves an immune system overreaction to substances, while heat-activated sensitivity is characterized by redness, flushing, and visible capillaries triggered by factors like intense exercise or sun exposure. Dry/dehydrated sensitivity often results from a compromised moisture barrier, leading to flaking, itchiness, and a burning sensation. Moreover, the Baumann Skin Typing System categorizes sensitive skin into four subtypes: acne, rosacea, stinging, and allergic [58]. Each subtype is reactive and prone to inflammation. Understanding one's specific sensitive skin type is imperative to select appropriate skincare products that effectively reduce inflammation. Additionally, research suggests that skin sensitivity can affect individuals across all skin types, and those with self-perceived sensitivity are more likely to experience objective stinging reactions [59]. This emphasizes the importance of considering both subjective perceptions and objective measures when evaluating skin sensitivity.

Downtime and Recovery Period

Following fractional CO2 laser treatment for acne scars, patients typically experience a downtime and recovery period lasting about a week on average [60]. During this time, patients are advised to avoid direct sunlight, opt for loose clothing, and abstain from strenuous activities that could potentially damage the treated skin. The procedure entails targeting specific columns of skin with the laser while leaving the surrounding skin unaffected, facilitating quicker healing and the development of new skin. Patients may encounter redness and sensitivity in the initial days post-treatment, which gradually diminishes as new skin emerges within a week. Moreover, punch excision of acne scars typically involves minimal downtime, with very fine sutures remaining on the skin for approximately five to seven days before patients can resume normal activities [61]. The recovery period for punch excision typically spans from a few days to a week, rendering it a relatively swift procedure with limited downtime.

Physician Expertise

Physician expertise in cancer care necessitates a multidisciplinary approach, wherein various specialists assume pivotal roles in diagnosis, treatment, and management. Oncologists specialize in diagnosing and treating cancer, while surgical oncologists are dedicated to performing surgeries aimed at removing tumors or affected body parts. Additionally, radiation oncologists focus on administering radiation therapy to treat cancer, and medical oncologists oversee general care, coordinate treatments, and manage chemotherapy, hormone therapy, targeted therapy, and immunotherapy [62]. Primary care physicians (PCPs) also play a crucial and active role in cancer patient management. They manage comorbid conditions, chronic pain, or depression, establish do-not-resuscitate status, and refer patients to hospice care when needed [63]. The involvement of PCPs in cancer care is significant, underscoring the importance of understanding the optimal interface between PCPs and oncologists to deliver coordinated cancer care effectively. When selecting a cancer specialist, several factors warrant consideration. These include their experience, training, board certification, and receptiveness to patient inquiries, ensuring a comprehensive and patient-centered approach to cancer treatment [62]. Patients can access high-quality care tailored to their needs and preferences by prioritizing these factors, enhancing the overall cancer treatment experience.

Future directions and emerging technologies

Advancements in Laser Technology

Laser technology is advancing rapidly, with innovations expanding the range of available wavelengths. Recent developments, such as green laser light absorption by nonferrous metals, are up-and-coming for applications in e-mobility [64]. Moreover, there's a noticeable trend towards miniaturizing lasers, including semiconductors and direct diode lasers, facilitating their integration into systems like cell phones and operating rooms. This miniaturization trend opens up new possibilities, such as laser-based scanning in autonomous vehicles [64]. Automation and artificial intelligence are increasingly integrated into laser technology, transforming processes across diverse fields such as plastic surgery. These advancements significantly enhance precision, quality, and productivity while unlocking new applications and improving overall outcomes [65]. Laser therapy devices are also undergoing optimization, streamlining, and automation to become more accessible, efficient, and non-invasive. Technologies like extended dye lives, increased wavelength capacities, and sophisticated interrogation systems contribute to enhanced precision and accuracy in laser treatments [65]. Looking ahead, the continuous growth in laser treatment advancements promises more efficient and effective care in the years to come. Clinicians must stay abreast of these evolving technologies to deliver optimal treatment for a broad spectrum of skin concerns [65]. By embracing these advancements, practitioners can ensure patients receive the most advanced and tailored laser treatments, improving outcomes and patient satisfaction.

Combination Therapies

Combination therapies for addressing acne scars entail employing multiple modalities to achieve optimal results. These treatments are tailored to target various types of acne scars, including atrophic scars, hypertrophic scars, icepick scars, rolling scars, and boxcar scars. By combining different approaches such as subcision, microneedling, chemical peels, and laser therapy, significant efficacy has been demonstrated in enhancing the appearance of acne scars [66-68]. For instance, one study showcased the effectiveness of a combination therapy incorporating subcision, microneedling, and a 15% trichloroacetic acid (TCA) peel in managing atrophic acne scars. The findings revealed substantial improvements in scar grades and high levels of patient satisfaction [67]. Similarly, another approach involving a triple combination of chemical reconstruction of skin scars (CROSS), subcision, and microneedling yielded consistent patient satisfaction and positive alterations in scar appearance [66]. These combination therapies offer a comprehensive approach to treating acne scars by addressing various aspects of scar tissue, collagen induction, skin texture enhancement, and scar release. The synergistic effects of integrating diverse treatments help bolster collagen production, facilitate skin resurfacing, and ultimately reduce scarring, culminating in improved outcomes for individuals grappling with acne scarring.

Potential Side Effect Mitigation

Careful patient selection, comprehensive evaluation, and diligent post-procedure care are paramount to mitigate potential side effects of laser resurfacing procedures, such as CO2 laser therapy. During the initial consultation, it is critical to assess factors that may predispose patients to adverse reactions, such as poor wound healing tendencies, a history of keloids, or hypertrophic scarring [29]. Patients with a background of easy tanning or extensive sun damage may necessitate tailored treatment approaches to minimize the risk of hyperpigmentation. Moreover, individuals with compromised immune systems should be closely monitored to mitigate the likelihood of post-procedure infections. Implementing appropriate postoperative care measures, including gentle cleansing, applying protective ointments, and administering antibiotics as needed, plays a pivotal role in promoting rapid healing and reducing the risk of infections [29]. By adhering to these preventive strategies and providing attentive care, side effects such as hypopigmentation, infection, or delayed healing can be significantly diminished, thereby ensuring a safer and more successful laser resurfacing experience for patients.

## Conclusions

In conclusion, this comprehensive review has shed light on the efficacy, safety, and comparative aspects of CO2 and Er:YAG laser therapy for treating acne scars. Both modalities have demonstrated effectiveness in enhancing the appearance of acne scars through collagen remodeling and tissue regeneration. While CO2 lasers are known for their ability to penetrate deeper tissues and potentially provide more significant improvement in severe acne scars, Er:YAG lasers offer a gentler approach with reduced risk of post-inflammatory hyperpigmentation. To optimize clinical practice, it is recommended that clinicians tailor treatment plans based on individual patient characteristics, educate patients about treatment expectations and post-treatment care, consider combination therapies for enhanced outcomes, and implement regular follow-up care to monitor progress and adjust treatment as necessary. However, further research is needed to assess long-term treatment outcomes, explore the efficacy and safety of laser therapy in diverse populations, investigate the synergistic effects of combination therapies, and evaluate emerging laser technologies for acne scar treatment. By addressing these research gaps, clinicians can continue to refine treatment strategies and improve outcomes for patients with acne scars.
